# 93. Leveraging Stewardship to Promote Narrower-spectrum Antibiotic Use for Low-risk AmpC Enterobacterales

**DOI:** 10.1093/ofid/ofad500.009

**Published:** 2023-11-27

**Authors:** Megan E Hardy, Rachel M Kenney, Robert Tibbetts, Anita Shallal, Michael Veve

**Affiliations:** WVU Medicine, Morgantown, WV; Henry Ford Hospital, Detroit, Michigan; Henry Ford Health, Detroit, Michigan; Henry Ford Health, Detroit, Michigan; Henry Ford Health, Detroit, Michigan

## Abstract

**Background:**

AmpC β-lactamases are associated with development of ceftriaxone (CRO) resistance despite *in vitro* susceptibility, but the risk of AmpC derepression is not equal among Enterobacterales. The purpose of this study was to evaluate the impact of an AmpC stewardship intervention on definitive treatment of low-risk Enterobacterales.

**Methods:**

IRB approved, single pre-test, post-test quasi-experiment with a non-equivalent dependent variable at a 5-hospital system. An AmpC stewardship intervention was implemented 7/22 and included education, removal of microbiology comments indicating potential for CRO resistance on therapy, and modification of a blood PCR comment for *Serratia marcescens* to recommend CRO. Inclusion: adults ≥ 18 years pre- (7/21-12/21) and post-intervention (7/22-12/22) who received ≥ 72 hours of inpatient definitive therapy and had non-urine cultures growing *S. marcescens*, *Providencia* spp., *Citrobacter koseri*, *C. amalonaticus*, *C. farmeri*, or *Morganella morganii*. Exclusion: infection with CRO resistant organisms. Primary outcome: proportion of patients who received definitive CRO therapy. Secondary outcomes at 30 days: retreatment for the same organism, development of CRO-resistant organisms, or *Clostridioides difficile* infection (CDI).

**Results:**

224 patients were included: 115 (51%) pre- and 109 (49%) post-intervention. **Table 1** describes patient, infection, and treatment characteristics. There were 79 (35%) patients with concurrent bacteremia. Definitive CRO therapy was prescribed more frequently after intervention 6 (5%) vs 72 (66%), *P*< 0.001. Median (IQR) total duration for pre- and post-groups (9 [7-17] vs 10 [7-18], *P*=0.46). After adjustment for intensive care, patients in the post-group were more likely to receive definitive CRO (adjOR, 35.4; 95%CI, 14.2-88.0) (**Table 2**). The proportion of patients who required retreatment was 18 (15%) and 11 (10%) for pre- and post-group patients (*P*=0.22). CRO resistance within 30 days occurred in 5 (4%) and 2 (2%) patients in the pre- and post-group (*P*=0.45).

**Table 1.**
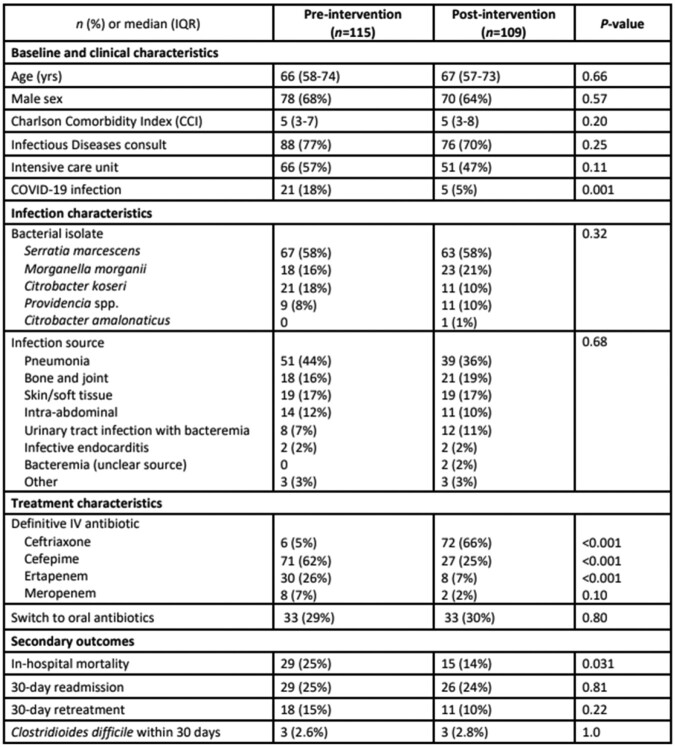
Patient, infection, and treatment characteristics

**Table 2.**
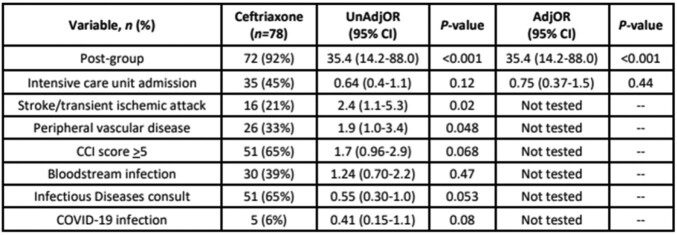
Variables associated with definitive CRO therapy

**Conclusion:**

An antimicrobial stewardship intervention was associated with increased CRO prescribing and similar patient outcomes for low-risk AmpC Enterobacterales.

**Disclosures:**

**Michael Veve, PharmD, MPH**, National Institutes of Health: Grant/Research Support|Paratek Pharmaceuticals: Grant/Research Support

